# Decreased Hepatic and Serum Levels of IL-10 Concur with Increased Lobular Inflammation in Morbidly Obese Patients

**DOI:** 10.3390/medicina60060862

**Published:** 2024-05-25

**Authors:** Helena Solleiro-Villavicencio, Lucía Angélica Méndez-García, Nydia A. Ocampo-Aguilera, Itzel Baltazar-Pérez, José A. Arreola-Miranda, José A. Aguayo-Guerrero, Ana Alfaro-Cruz, Antonio González-Chávez, Miguel A. Fonseca-Sánchez, José Manuel Fragoso, Galileo Escobedo

**Affiliations:** 1 Posgrado en Ciencias Genómicas, Universidad Autónoma de la Ciudad de México, Mexico City 03100, Mexico; helena.solleiro@uacm.edu.mx; 2 Laboratory of Immunometabolism, Research Division, General Hospital of Mexico “Dr. Eduardo Liceaga”, Mexico City 06726, Mexico; lucia.angelica86@gmail.com (L.A.M.-G.); angelica.ocampo@alumnos.uacm.edu.mx (N.A.O.-A.); itzel.bp.050997@gmail.com (I.B.-P.); alfonso.arreola@estudiante.uacm.edu.mx (J.A.A.-M.); jose.aguayo01@iest.edu.mx (J.A.A.-G.); 3 Pathological Anatomy Department, General Hospital of Mexico “Dr. Eduardo Liceaga”, Mexico City 06726, Mexico; analfaro@yahoo.com; 4 Clínica de Atención Integral para Pacientes con Diabetes y Obesidad (CAIDO), General Hospital of Mexico “Dr. Eduardo Liceaga”, Mexico City 06726, Mexico; antoniogonzalezchavez51@gmail.com; 5 Research Division, General Hospital of Mexico “Dr. Eduardo Liceaga”, Mexico City 06726, Mexico; mfonseca_79@hotmail.com; 6 Department of Molecular Biology, Instituto Nacional de Cardiología Ignacio Chávez, Juan Badiano 1, Sección XVI, Tlalpan, Mexico City 14080, Mexico; mfragoso1275@yahoo.com.mx

**Keywords:** IL-10, lobular inflammation, simple steatosis, NASH, morbid obesity, liver biopsy

## Abstract

*Background and Objectives*: Non-alcoholic fatty liver disease (NAFLD) is associated with obesity and ranges from simple steatosis to non-alcoholic steatohepatitis (NASH), fibrosis, cirrhosis, and hepatocellular carcinoma. Accumulating evidence in animal models suggests that loss of interleukin-10 (IL-10) anti-inflammatory actions might contribute to lobular inflammation, considered one of the first steps toward NASH development. However, the role of IL-10 in lobular inflammation remains poorly explored in humans. We examined mRNA and protein levels of IL-10 in liver biopsies and serum samples from morbidly obese patients, investigating the relationship between IL-10 and lobular inflammation degree. *Materials and Methods*: We prospectively enrolled morbidly obese patients of both sexes, assessing the lobular inflammation grade by the Brunt scoring system to categorize participants into mild (*n* = 7), moderate (*n* = 19), or severe (*n* = 13) lobular inflammation groups. We quantified the hepatic mRNA expression of IL-10 by quantitative polymerase chain reaction and protein IL-10 levels in liver and serum samples by Luminex Assay. We estimated statistical differences by one-way analysis of variance (ANOVA) and Tukey’s multiple comparison test. *Results*: The hepatic expression of IL-10 significantly diminished in patients with severe lobular inflammation compared with the moderate lobular inflammation group (*p* = 0.01). The hepatic IL-10 protein levels decreased in patients with moderate or severe lobular inflammation compared with the mild lobular inflammation group (*p* = 0.008 and *p* = 0.0008, respectively). In circulation, IL-10 also significantly decreased in subjects with moderate or severe lobular inflammation compared with the mild lobular inflammation group (*p* = 0.005 and *p* < 0.0001, respectively). *Conclusions*: In liver biopsies and serum samples of morbidly obese patients, the protein levels of IL-10 progressively decrease as lobular inflammation increases, supporting the hypothesis that lobular inflammation develops because of the loss of the IL-10-mediated anti-inflammatory counterbalance.

## 1. Introduction

Non-alcoholic fatty liver disease (NAFLD) is a highly prevalent hepatic disorder worldwide, characterized by excess build-up of lipids in the liver without disproportionate alcohol consumption [1]. NAFLD encompasses a spectrum of conditions ranging from simple steatosis to non-alcoholic steatohepatitis (NASH), which involves inflammation, hepatocyte injury, and liver fibrosis [2]. Still, hepatologists can only belatedly suspect NASH after finding abnormally high-liver-function tests in patients who often show obesity and metabolic syndrome, increasing the risk of aggravating liver disease until NASH diagnosis by liver biopsy [3]. NASH can progress to fibrosis, cirrhosis, and even hepatocellular carcinoma (HCC) through an intricated network of interactions among metabolic, genetic, and immune factors, where lobular inflammation plays a central role [4].

Lobular inflammation consists of immune cell aggregates in the hepatic lobules, primarily Kupffer cells, monocyte-derived macrophages (MDMs), and neutrophils, and is a crucial histopathological feature in NASH diagnosis [5,6]. Kupffer cells and MDMs invading the hepatic lobules can synthesize proinflammatory molecules that counteract the actions of a critical factor in charge of attenuating lobular inflammation, interleukin-10 (IL-10) [7,8]. IL-10 is an anti-inflammatory cytokine that exerts immunosuppressive effects by inhibiting proinflammatory cytokine production and modulating immune cell function [9,10,11]. Numerous research teams have consistently reported that IL-10 plays protective roles in several liver conditions, such as viral hepatitis, autoimmune liver illness, and alcoholic liver disease [12,13,14]. A growing body of evidence, mostly in animal models, shows that progressive loss of IL-10 anti-inflammatory actions leads to worsening liver pathology, including fibrosis, hepatocyte apoptosis, and cholangiocyte injury [15,16]. However, the role of IL-10 in lobular inflammation as one of the first crucial steps in NASH development remains poorly explored in humans.

Herein, we examined mRNA and protein levels of IL-10 in liver biopsies and serum samples from morbidly obese patients, investigating whether IL-10 can help identify the lobular inflammation grade in subjects with no apparent compromise in liver function.

## 2. Materials and Methods

### 2.1. Patients

We conducted a prospective, cross-sectional study, enrolling *n* = 39 morbidly obese patients of both sexes who met the selection criteria and were scheduled for elective Roux-en-Y gastric bypass in the Clinic for Patients with Obesity and Diabetes and the General Surgery Department of General Hospital of Mexico from February 2022 to September 2023. All enrolled patients signed the informed consent letter previously approved by General Hospital of Mexico’s Ethics Committee with the registration of the project number DI/16/UME/05/048, agreeing to donate 6 mL of venous blood before the surgery and a 3 g liver sample during surgery. We excluded from the study patients with previous diagnoses of endocrine syndromes, infectious diseases including a confirmatory test for COVID-19 or influenza by quantitative polymerase chain reaction (qPCR), autoimmune disorders, and cancer, and individuals under any immunomodulatory drug treatment in the previous three months. We conducted the study in rigorous observance of the principles of the Declaration of Helsinki in 1964 and its subsequent amendment in 2013.

### 2.2. Registration of Demographic, Clinical, and Biochemical Data

We registered demographic, clinical, and biochemical data in all enrolled patients, including full name, clinical record number, sex, age, body mass index (BMI), the prevalence of type 2 diabetes (T2D) and systemic arterial hypertension (SAH), coagulation parameters (including prothrombin time (PT), international normalized ratio (INR), and activated partial thromboplastin time (aPTT)), fasting blood glucose, triglycerides, total cholesterol, low-density lipoproteins (LDL-C), high-density lipoproteins (HDL-C), urea, creatinine, uric acid, hemoglobin, alanine aminotransferase (ALT), aspartate aminotransferase (AST), gamma-glutamyl transferase (GGT), alkaline phosphatase (AP), total bilirubin (TB), direct bilirubin (DB), and indirect bilirubin (IB). We measured all laboratory variables by using the Beckman Coulter DxC 700 AU Chemistry Analyzer (Beckman Coulter Inc., Brea, CA, USA) and the BCS^®^ XP System (Siemens Healthcare GmbH, Erlangen, Germany).

### 2.3. Liver Histology and Lobular Inflammation Grading

We fixed and embedded liver specimens in paraffin for histological processing, cutting slices around 4 μm thick by using a microtome (Leica Biosystems, Deer Park, IL, USA). We stained slices with hematoxylin–eosin to visualize the cell nuclei and the cytoplasmic components of hepatic cells, examining the areas between the hepatic lobules to score lobular inflammation. We used the Brunt scoring system to quantify lobular inflammation, assessing the number of inflammatory foci at the hepatic lobules per 20× fields as follows: mild lobular inflammation, <2 inflammatory foci/20× fields; moderate lobular inflammation, 2–4 inflammatory foci/20× fields; severe lobular inflammation, >4 inflammatory foci/20× fields [17]. We also used the Brunt scoring system to measure steatosis degree and stage of fibrosis by liver Picro-Sirius red staining and Masson’s trichrome staining, respectively.

### 2.4. Measurement of IL-10 Levels in Liver and Serum

We quantified the hepatic mRNA gene expression of IL-10 by qPCR as follows: We placed liver samples in TRIzol reagent (Invitrogen, Carlsbad, CA, USA) for posterior RNA isolation. We used total RNA for reverse transcription by using the M-MLV retrotranscriptase system and dT primer (Invitrogen, Carlsbad, CA, USA). Then, we used 200 ng/μL cDNA for qPCR by using SsoAdvanced Universal SYBR Green Supermix (Bio-Rad Laboratories, Inc., Hercules, CA, USA) and 500 nM human IL-10 specific primer. We used the 18S-ribosomal RNA primer as a housekeeping gene for ΔΔCT calculation and normalization, expressing results as fold change. We measured IL-10 protein levels by disrupting the liver tissue in 500 mM Tris-HCl and protease inhibitor cocktail (Merck, Darmstadt, Germany). After a centrifugation step at 20,800× *g*/8 °C for 15 min, we recovered the supernatant and quantified the protein concentration by the Bradford Protein Assay (Bio-Rad Laboratories, Inc., Hercules, CA, USA) at 595 nm. Then, we used the Human IL-10 Magnetic Luminex^®^ Performance Assay Kit (R&D Systems, Minneapolis, MN, USA) for detecting IL-10, generating a standard curve with the pre-mixed standards included in the kit to calculate cytokine concentration in pg/mL. To measure IL-10 serum levels, we collected 6 mL of venous blood in golden cap tubes (Vacutainer; BD Diagnostics, Franklin Lakes, NJ, USA). After centrifuging samples at 1800× *g* for 15 min at room temperature, we isolated serum fractions for determining IL-10 levels with the Human IL-10 Magnetic Luminex^®^ Performance Assay Kit (R&D Systems, Minneapolis, MN, USA), calculating cytokine concentration in pg/mL through a standard curve.

### 2.5. Statistics

After assessing the normality of data by the Shapiro–Wilk test, we compared the numerical variables by one-way analysis of variance (ANOVA) followed by Tukey’s multiple comparison test, expressing the values as means ± standard deviation. We analyzed categorical variables by the chi-squared test and showed the values as absolute values, means ± standard deviation, or percentages. We adjusted differences in IL-10 values among mild, moderate, and severe lobular inflammation groups by confounding variables with multiple regression analysis with the terminal R 3.5.1. We considered differences significant when *p* < 0.05, using GraphPad Prism 7 software.

## 3. Results

We enrolled 39 patients with morbid obesity, finding 7 subjects with mild lobular inflammation, 19 individuals exhibiting moderate lobular inflammation, and 13 patients with severe lobular inflammation (Table 1). There were no differences among subjects who developed mild, moderate, or severe lobular inflammation for sex proportion, age, and BMI (Table 1). The most prevalent comorbidities found in our study population were T2D and SAH, with no differences among patients showing a mild, moderate, or severe degree of lobular inflammation (Table 1).

Results from the one-way ANOVA and Tukey’s multiple comparison tests indicate no significant changes among morbidly obese patients with mild, moderate, or severe lobular inflammation for blood glucose and lipid profile, including total cholesterol, HDL-C, LDL-C, and triglycerides (Table 2). In the same sense, there were no differences in our study population in urea, creatinine, nor uric acid (Table 2). Coagulation tests also revealed no differences among subjects with mild, moderate, and severe lobular inflammation for PT, INR, aPTT, hemoglobin, and platelet number (Table 2). Notably, the leukocyte number increased only in patients with moderate lobular inflammation compared with participants exhibiting mild lobular inflammation (*p* = 0.008) (Table 2). Except for GGT values, which increased in patients with severe lobular inflammation compared with subjects showing mild lobular inflammation (*p* = 0.029), there were no differences in the study population for liver function tests, including ALP, AST, ALT, total bilirubin, and albumin, among others (Table 2).

**Figure 1:**
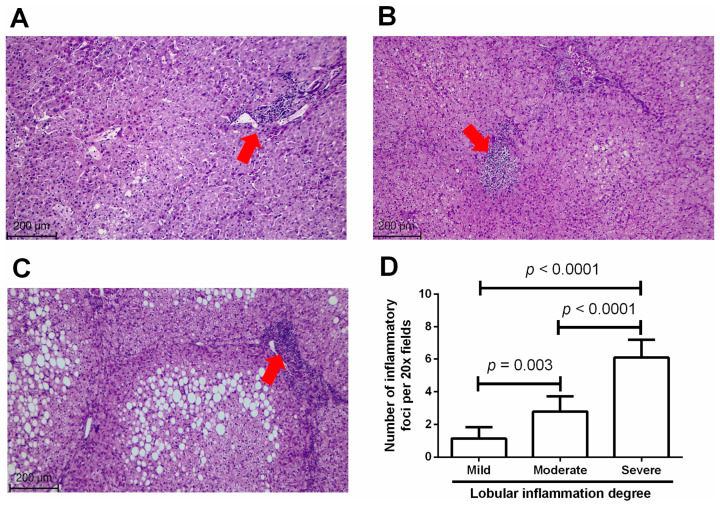
Histologic examination and grading of liver specimens. We stained 4 μm thick slices of liver tissue with hematoxylin–eosin, examining the areas between the hepatic lobules to score lobular inflammation according to the Brunt scoring system as follows: (**A**) mild lobular inflammation, <2 inflammatory foci/20× fields; (**B**) moderate lobular inflammation, 2–4 inflammatory foci/20× fields; (**C**) severe lobular inflammation, >4 inflammatory foci/20× fields. Red arrows indicate the presence of inflammatory foci. Scale bars show the 200 μm magnification at which we acquired the microphotographs. (**D**) After quantifying the number of inflammatory foci, we compared data by one-way ANOVA followed by Tukey’s multiple comparison test, considering a *p* < 0.05 significant.

Figure 1 shows representative microphotographs illustrating several histopathologic features in the liver of morbidly obese patients, such as inflammatory infiltrate at the hepatic lobules, lipid droplet deposition, and fibrosis. Subjects within the mild lobular inflammation group displayed scant inflammatory infiltrate around the hepatic lobules with no apparent lipid droplet formation (Figure 1A). On the contrary, individuals belonging to the moderate lobular inflammation group showed an increased number of inflammatory foci at the hepatic lobules accompanied by a scarce number of lipid droplets (Figure 1B). Nevertheless, patients within the severe lobular inflammation exhibited a considerable increase in the number of inflammatory cells with abundant lipid droplet accumulation (Figure 1C). We found histologic evidence of scant fibrosis only in one participant of the severe lobular inflammation group but not in subjects showing mild or moderate lobular inflammation. There was no histologic sign showing some level of hepatocyte ballooning associated with the lobular inflammation degree or fat infiltration (Figure 1A–C). After quantifying the number of inflammatory foci at the hepatic lobules, we found that subjects within the mild lobular inflammation group showed 1.1 ± 0.6 inflammatory foci per 20× fields (Figure 1D). In contrast, patients with moderate or severe lobular inflammation exhibited 2.8 ± 0.9 and 6.1 ± 1.1 inflammatory foci around the hepatic lobules per 20× fields, respectively, highlighting a notable increase with respect to that found in individuals with mild lobular inflammation (*p* < 0.0001 in both cases) (Figure 1D).

**Figure 2:**
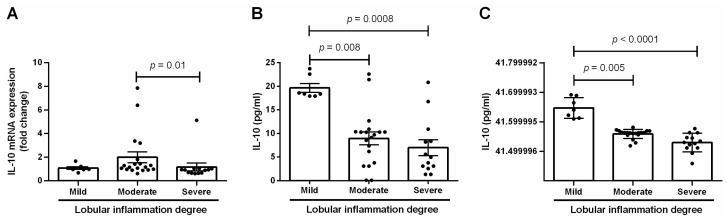
Quantification of IL-10 levels in liver tissue and serum. (**A**) IL-10 mRNA expression in liver specimens from morbidly obese patients with several degrees of lobular inflammation. (**B**) IL-10 protein levels in hepatic tissue from patients with different grades of lobular inflammation. (**C**) IL-10 serum values in liver samples from morbidly obese patients with several degrees of lobular inflammation. We compared data by one-way ANOVA followed by Tukey’s multiple comparison test, considering a *p* < 0.05 significant. IL-10, interleukin 10.

The qPCR analyses revealed that patients with severe lobular inflammation displayed a significant decrease in the hepatic mRNA expression of IL-10 compared with individuals showing moderate lobular inflammation (*p* = 0.01) (Figure 2A). In the same sense, subjects exhibiting moderate lobular inflammation showed an apparent 2-fold decrease in IL-10 protein levels compared with that found in individuals with mild lobular inflammation (*p* = 0.008) (Figure 2B). Notably, patients belonging to the severe lobular inflammation group exhibited an evident 2.8-fold reduction in IL-10 protein values compared with subjects within the mild lobular inflammation group (*p* = 0.0008) (Figure 2B). It is worth mentioning that the circulating levels of IL-10 displayed the same behavior as IL-10 mRNA and protein values in hepatic tissue. Specifically, individuals in the moderate lobular inflammation group showed a significant 11 percent reduction in the serum IL-10 levels compared with subjects with mild lobular inflammation (*p* = 0.005) (Figure 2C). However, patients with severe lobular inflammation exhibited an evident 20 percent diminution in the circulating values of IL-10 compared with subjects showing mild lobular inflammation (*p* < 0.0001) (Figure 2C). Differences in both mRNA and protein levels of IL-10 among mild, moderate, or severe lobular inflammation subjects remained unaltered after adjusting for confounding variables, including age, sex, the timing of obesity, T2D, and SAH.

## 4. Discussion

Accumulating evidence shows that lobular inflammation is one of the critical steps that trigger the inflammatory events accompanying NASH development in obese patients with liver steatosis [18,19,20]. Most authors concur that excessive fat accumulation in the liver instigates lipid peroxidation, which leads to free-radical production, including reactive oxygen species (ROS) such as superoxide anion (O_2_^−^) and hydroperoxide (O_2_H) [21,22,23]. O_2_^−^ and O_2_H induce liver damage by promoting hepatocyte apoptosis and necrotic cell death, initiating an inflammatory cascade leading to immune cell infiltration and proinflammatory cytokine release into the hepatic lobules, which perpetuate liver damage and inflammation [24,25]. In this scenario, the contribution of lipid accumulation and immune activity to developing lobular inflammation seems clear; however, the possible role of the anti-inflammatory response mediated by immunomodulatory cytokines such as IL-10 is still uncertain. Herein, we used a complementary experimental strategy in liver biopsies and serum samples from morbidly obese patients and found that IL-10 progressively decreases as lobular inflammation increases, even though hepatocyte ballooning and fibrosis are still absent, and ALT and AST remain unaltered.

The usefulness of liver function tests to detect hepatic alterations and diagnose liver disease has been proven since the half of the 20th century [26]. Alteration in liver enzymes is a clinical guide for diagnosing liver disorders, including bile duct obstructions, hepatitis due to several causes, autoimmune cholestatic diseases, liver toxicity, cirrhosis, and HCC, among others [27,28]. However, the diagnosis of NASH is still a challenge for hepatologists, because testing liver enzymes does not seem to assess the disease accurately, and liver biopsy is still considered the only test able to diagnose it [29]. In this regard, several studies have reported a significant proportion of patients with liver steatosis or NASH exhibiting normal levels of liver function tests [30,31]. For instance, P. Mofard et al., 2003, reported that among obese individuals with biopsy-proven steatosis, approximately 40% had normal ALT levels [32]. Another study informed that nearly 50% of obese patients with biopsy-proven NASH showed normal ALT levels with no difference in the AST/ALT ratio compared with obese subjects without histologically proven NASH [33]. Our data concur with previous information, indicating normal ALT and AST levels in morbidly obese patients exhibiting mild, moderate, or severe lobular inflammation. We may attribute this finding to the fact that patients enrolled in this study are at a very early stage of liver damage, with no histologic evidence of hepatocyte ballooning or fibrosis that can be associated with liver enzyme alteration [34,35]. This information highlights the urgency of finding novel markers that help detect lobular inflammation when fibrosis and even liver enzymes remain steady, thus preventing NASH development and liver compromise.

An exciting phenomenon captured in our study involves the GGT levels, which were elevated only in morbidly obese patients with severe lobular inflammation compared with subjects with mild lobular inflammation. In this sense, a study conducted in a large cohort of patients from Bangladesh reported that GGT was the only liver enzyme that better predicted NASH, with a sensitivity of 45% and a specificity of 68% [36]. Moreover, G. Feng et al., 2020 stated that among all liver function parameters, GGT is better associated with NASH progression regardless of the BMI of patients, while another research team demonstrated that GGT predicts NASH with an area under the receiver operating characteristic (ROC) curve of 0.68 [37,38]. This evidence supports our data showing that GGT is the only liver enzyme that is altered in response to lobular inflammation, opening an exciting avenue to assess GGT as an early predictor for NASH in morbidly obese patients.

For many years, excessive weight gain observed during obesity progression was associated directly with simple steatosis, NASH, and liver damage [39]. However, E.T. Poehlman and colleagues described a group of obese subjects who did not show the metabolic disturbances typically attributed to excessive weight gain, such as insulin resistance and hyperglycemia, adopting for the first time the term metabolically healthy obesity [40]. The concept of metabolically healthy obesity was controversial when discussing the evidence regarding liver disorders in obese patients. For instance, Helda Tutunchi et al., 2021 reported that metabolically unhealthy obese subjects have a higher risk for simple steatosis and fibrosis than metabolically healthy obese individuals [41]. In contrast, Ji Hye Huh et al., 2017 stated that the occurrence of simple steatosis and fibrosis is similar in metabolically unhealthy obese subjects compared with metabolically healthy patients [42]. In this study, we could only detect liver biopsy-proven lipid infiltration in 80% of morbidly obese participants. Contrary to expectations, almost 20% of subjects with morbid obesity did not have histologic evidence of simple steatosis. Furthermore, despite all studied subjects having morbid obesity, which is considered the highest state of body weight and cardiovascular risk, not all participants showed the same grade of biopsy-proven lobular inflammation in the liver. These findings underscore the variability in simple steatosis and inflammation among obese individuals, even after adjusting for potentially confounding variables such as age, sex, the timing of obesity, T2D, and SAH. Therefore, it is crucial to find novel markers that could help identify and monitor individuals at higher risk of developing lobular inflammation independently of the degree of obesity, such as IL-10.

In a previous study, our research team reported that IL-10 serum values progressively reduced as the steatosis grade increased in morbidly obese patients when estimated by abdominal ultrasound imaging [43]. A similar study found that IL-10 circulating levels decreased in overweight or obese patients with simple steatosis, reaching the most substantial diminution when steatosis was accompanied by NASH [44]. What is more, both studies outlined, by different experimental strategies, that IL-10 reduction was hallmarked by an increase in the serum levels of tumor necrosis factor-alpha (TNF-alpha), a proinflammatory cytokine with key actions in the liver inflammation of diverse etiologies [45,46,47]. This evidence suggests the existence of a hepatic microenvironment resulting from a balance between proinflammatory and anti-inflammatory pathways, where the absence of IL-10 might break the equilibrium, contributing to lobular inflammation and NASH. Once released, IL-10 binds to its cognate receptor, IL-10R, located in the membranal surface of monocytes, macrophages, dendritic cells (DCs), natural killer (NK) cells, T lymphocytes, B lymphocytes, and Kupffer cells, among others [48]. The interaction between IL-10 and IL-10R instigates the Janus kinase (JAK) signal transducer and activator of transcription 3 (STAT3) cascade, where phosphorylated STAT3 can translocate to the nucleus and induce the expression of crucial anti-inflammatory genes [49]. Among the most critical anti-inflammatory genes induced by IL-10 are Bcl3, Etv3, and ABIN-3, which impair the translocation and activity of nuclear factor kappa B (NFκB), a transcription factor essential to TNF-alpha expression. At the same time, IL-10-induced SHIP-1 and Zfp36 expression can inhibit TNF-alpha translation directly, thus resulting in the abrogation of the TNF-alpha-promoted proinflammatory events in the target tissue, as in the liver [48].

Due to lipid peroxidation and ROS production, hepatocytes release TNF-alpha, which stimulates SREBP-1c expression, prolonging de novo lipogenesis and hepatic fat accumulation [50]. Moreover, TNF-alpha orchestrates the production of IL-1 beta, IL-6, IL-8, chemokines, and adhesion molecules, which results in the recruitment of lymphocytes, monocytes, and neutrophils and the activation of tissue-resident macrophages, the Kupffer cells [51,52,53,54]. Thus, the TNF-alpha-mediated cellular immune response magnifies and perpetuates the proinflammatory microenvironment in the hepatic lobule, which represents the peripheral immune cell’s primary entrance point to the liver parenchyma [55]. Based on our findings, we speculate that in the absence of IL-10, TNF-alpha may exert all these inflammatory actions without any anti-inflammatory counterbalance, supporting the data that suggest a causal relationship between reduced IL-10 production and increased lobular inflammation. However, we still need to conduct further experiments to measure TNF-alpha and IL-10 simultaneously in liver biopsies to test the hypothesis that the loss of proinflammatory and anti-inflammatory balance in simple steatosis is crucial to lobular inflammation and NASH.

This study has several limitations, including the cross-sectional nature of the study, which does not allow us to establish a clear cause-and-effect relation between IL-10 levels and lobular inflammation until additional research is performed. Moreover, although the sample size was large enough to estimate statistically significant differences, increasing the number of study subjects may enlarge the probability of finding liver biopsies without any degree of lobular inflammation to examine IL-10 production on that condition. Expanding the sample size will also allow us to calculate the area under the ROC curves for the serum levels of IL-10 to determine its utility as a predictor for lobular inflammation. Finally, complete immune cell characterization in the liver is still pending to find the cellular source of IL-10 and other cytokines with prominent proinflammatory and anti-inflammatory activities in lobular inflammation and NASH.

## 5. Conclusions

In the liver biopsies of morbidly obese patients, IL-10 protein levels progressively decreased as lobular inflammation increased. In line with this finding, IL-10 serum values also gradually decreased by the same proportion as lobular inflammation increased, even though most liver enzymes remained unaltered and after adjusting for confounding variables, such as age, sex, T2D, and SAH. Our data support the hypothesis that lobular inflammation develops because of the loss of the IL-10-mediated anti-inflammatory counterbalance, which might polarize the liver towards a proinflammatory microenvironment that leads to lobular inflammation and NASH. We must conduct further studies to assess the ability of IL-10 serum levels to be used as a potential marker of lobular inflammation development in morbidly obese patients who still show no biopsy-proven liver damage, including hepatocyte ballooning and fibrosis, and altered liver function tests.

## Figures and Tables

**Table 1:** Demographic and clinical characteristics of the study participants.

	Lobular Inflammation Degree			
*n* = 39	Mild *n* = 7	Moderate *n* = 19	Severe *n* = 13	*p*-Value
Women/men	5/2	16/3	10/3	0.639
Age (years)	38.5 ± 7.7	40.0 ± 10.5	37.3 ± 10.3	0.541
BMI (kg/m^2^)	36.4 ± 5.9	40.6 ± 12.2	39.1 ± 7.1	0.703
T2D prevalence (%)	28.5	26.3	15.3	0.062
SAH prevalence (%)	28.5	42.1	30.7	0.075

We present data as absolute values, means ± standard deviation, or percentages. We used the chi-squared test to estimate differences for women/men proportion and prevalence of T2D and SAH. We compared age and BMI by one-way ANOVA. We considered differences significant when *p* < 0.05, using GraphPad Prism 7 software. BMI, body mass index; T2D, type 2 diabetes; SAH, systemic arterial hypertension.

**Table 2:** Biochemical parameters of the study participants.

	Lobular Inflammation Degree			
*n* = 39	Mild ^a^ *n* = 7	Moderate ^b^ *n* = 19	Severe ^c^ *n* = 13	*p*-Value
Blood glucose (mg/dL)	110.7 ± 34.4	101.4 ± 18.9	106.0 ± 14.6	0.450
Total cholesterol (mg/dL)	186.6 ± 42.5	161.8 ± 26.5	181.6 ± 35.5	0.154
HDL-C (mg/dL)	49.6 ± 19.9	42.0 ± 7.3	39.5 ± 14.8	0.327
LDL-C (mg/dL)	126.8 ± 41.4	101.5 ± 21.3	125.3 ± 28.4	0.061
Triglycerides (mg/dL)	130.6 ± 45.2	151.4 ± 50.7	182.0 ± 80.9	0.236
Urea (mg/dL)	35.8 ± 11.0	32.8 ± 9.4	31.2 ± 7.9	0.639
Creatinine (mg/dL)	0.9 ± 0.1	0.7 ± 0.1	0.7 ± 0.1	0.073
Uric acid (mg/dL)	6.7 ± 1.2	6.9 ± 2.9	5.8 ± 1.1	0.513
PT (s)	13.7 ± 5.9	11.1 ± 0.8	13.3 ± 4.2	0.218
INR (a.u.)	1.1 ± 0.5	0.9 ± 0.0	0.9 ± 0.1	0.231
aPTT (s)	26.4 ± 5.8	23.2 ± 6.1	23.2 ± 4.7	0.565
Hemoglobin (g/dL)	14.3 ± 0.6	15.0 ± 1.4	15.0 ± 1.4	0.590
Platelets (×10^3^/μL)	303.8 ± 58.1	307.0 ± 75.3	293.4 ± 80.1	0.388
Leukocytes (×10^3^/μL)	6.5 ± 1.1	9.0 ± 1.6	8.7 ± 1.6	0.008 ^a vs. b^
ALP (IU/L)	81.8 ± 30.6	93.0 ± 56.9	88.7 ± 24.9	0.881
GGT (IU/L)	15.5 ± 6.4	29.9 ± 15.1	35.2 ± 10.2	0.029 ^a vs. c^
AST (IU/L)	21.7 ± 8.2	25.7 ± 12.0	24.1 ± 8.2	0.782
ALT (IU/L)	25.2 ± 17.2	29.1 ± 15.9	33.0 ± 13.9	0.670
Total bilirubin (mg/dL)	0.7 ± 0.3	0.6 ± 0.3	0.6 ± 0.3	0.785
Direct bilirubin (mg/dL)	0.2 ± 0.1	0.1 ± 0.0	0.1 ± 0.0	0.718
Indirect bilirubin (mg/dL)	0.5 ± 0.2	0.5 ± 0.1	0.5 ± 0.3	0.716
Albumin (g/dL)	4.3 ± 0.2	4.3 ± 0.2	4.3 ± 0.3	0.942

We present data as means ± standard deviation, estimating significant differences by one-way ANOVA. We considered differences significant when *p* < 0.05, using GraphPad Prism 7 software. When indicated, superscripts a, b, or c show *p*-values resulting from comparing two specific groups by Tukey’s multiple comparison test. HDL-C, high-density lipoproteins; LDL-C, low-density lipoproteins; PT, prothrombin time; INR, international normalized ratio; aPTT, activated partial thromboplastin time; ALP, alkaline phosphatase; GGT, gamma-glutamyl transferase; AST, aspartate aminotransferase; ALT, alanine aminotransferase; a.u., arbitrary units.

## Data Availability

Data are available upon request.
